# Supplementary material for: Taste Masking of Promethazine Hydrochloride Using l-Arginine Polyamide-Based Nanocapsules
Source: Molecules. 2023 Jan 11;28(2):748. doi: 10.3390/molecules28020748 (PMC9865149; doi:10.3390/molecules28020748)
Supplement: Supplementary file 1 [file molecules-28-00748-s001.zip › molecules-2054900-supplementary.pdf]

Supplementary Materials

Taste Masking of Promethazine Hydrochloride Using L-Arginine Polyamide-Based Nanocapsules

Hamad S. Alyami [1], Dalia Khalil Ali [2], Qais Jarrar [3], Abdolelah Jaradat [3], Hadeel Aburass [3], Abdul Aleem Mohammed [1], Mohammad H. Alyami [1], Alhassan H. Aodah [4] and Eman Zmaily Dahmash [5,*]

[1] Department of Pharmaceutics, College of Pharmacy, Najran University, Najran 55461, Saudi Arabia
[2] Department of Physiotherapy, Faculty of Allied Medical Sciences, Isra University, Amman 1162, Jordan;
[3] Department of Applied Pharmaceutical Sciences and Clinical Pharmacy, Faculty of Pharmacy, Isra University, Amman 11622, Jordan
[4] National Center of Biotechnology, Life Science & Environment Research Institute, King Abdulaziz City for Science and Technology, Riyadh, 11442, Saudi Arabia
[5] Department of Chemical and Pharmaceutical Sciences, School of Life Sciences, Pharmacy and Chemistry, Kingston University London, Kingston upon Thames KT1 2EE, UK
* Correspondence: e.dahmash@kingston.ac.uk; Tel.: +44-7542329215

Section S1

Representative HPLC chromatograms

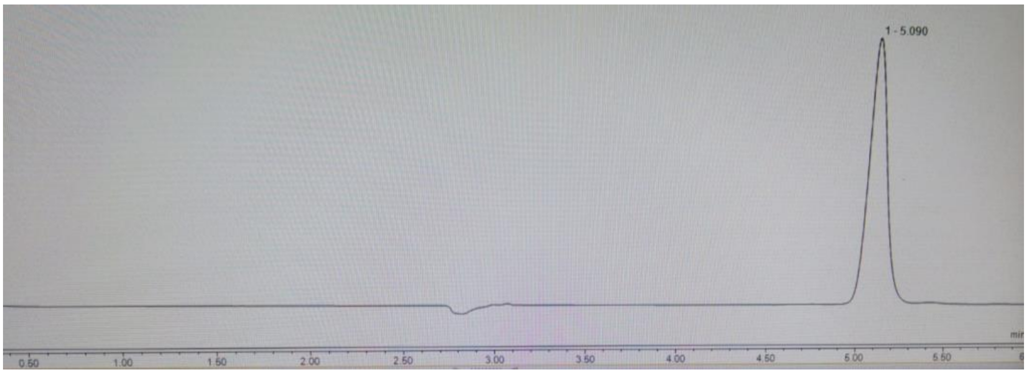

Figure S1. Representative HPLC chromatograms of PMZ with retention time at 5.09 minutes

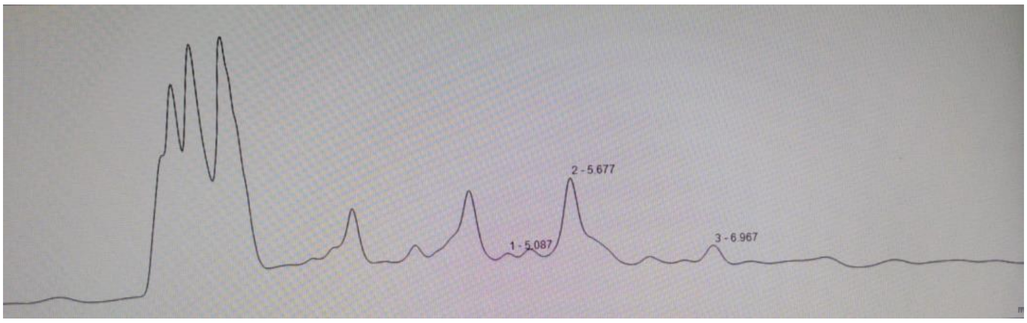

Figure S2. Representative HPLC chromatograms of the polymer highlighting no interference at the retention time of PMZ

Section S2

Release Kinetics of PMZ from PMZ/ Arg-PA NCs

The release results of PMZ from the formula PMZ/ Arg PA NCs were modelled using the commonly used mathematical models and results are presented in Figures S3-S7

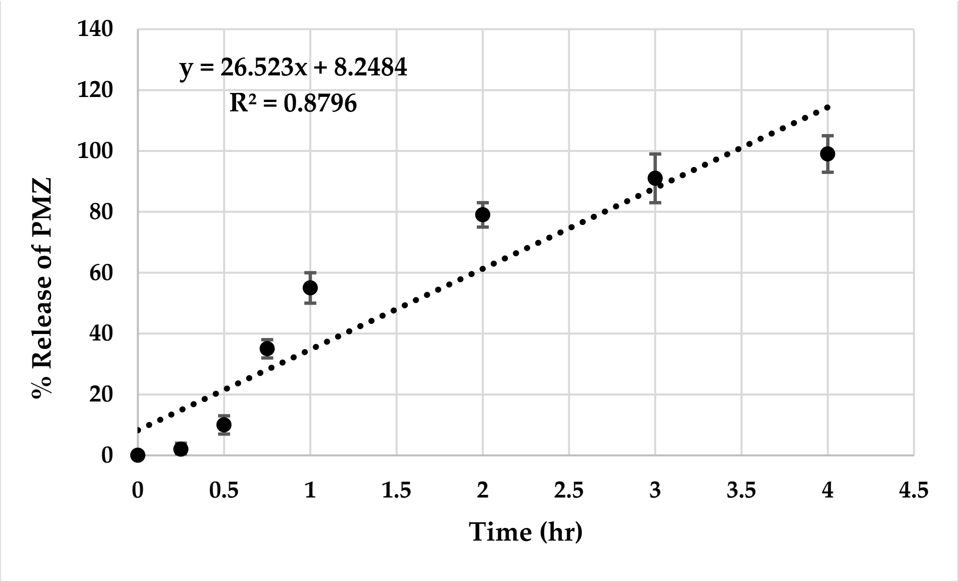

Figure S3. Linear regression of the Zero order kinetic mathematical model of PMZ from PMZ/Arg-PA NCs (F6). (R^2 is the coefficient of determination)

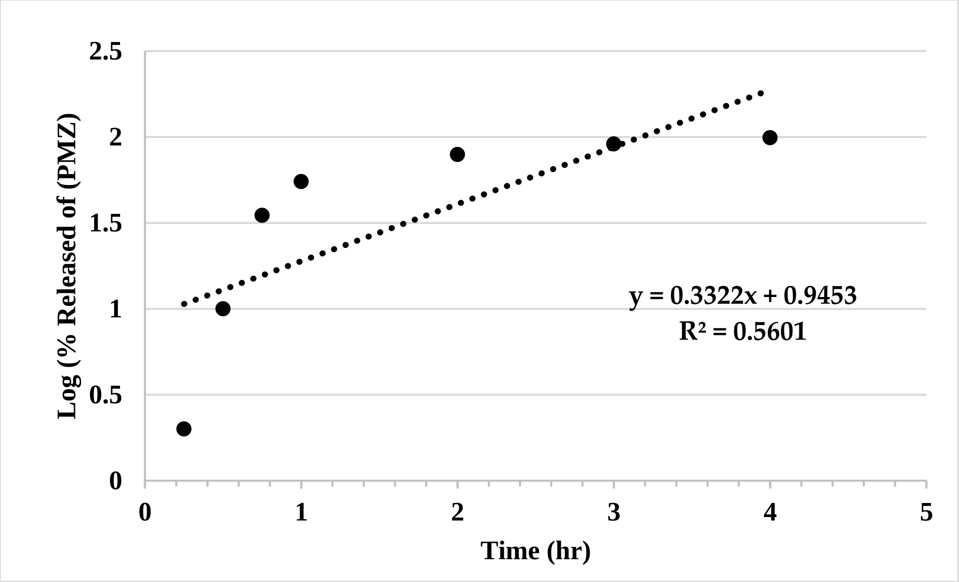

Figure S4. Linear regression of the First order kinetic mathematical model of PMZ from PMZ/Arg-PA NCs (F6). (R^2 is the coefficient of determination)

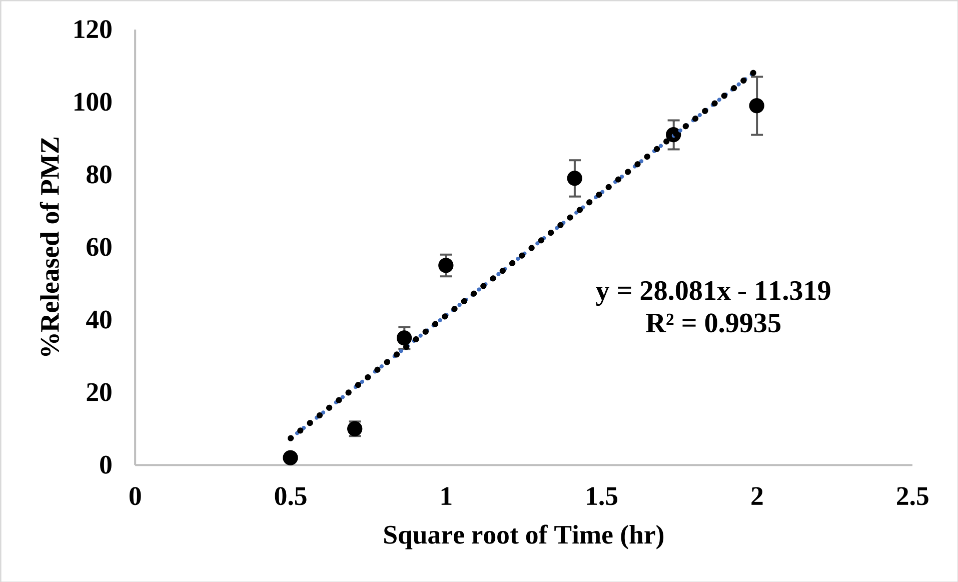

Figure S5. Linear regression of the Higuchi kinetic mathematical model of PMZ from PMZ/Arg-PA NCs (F6). (R^2 is the coefficient of determination)

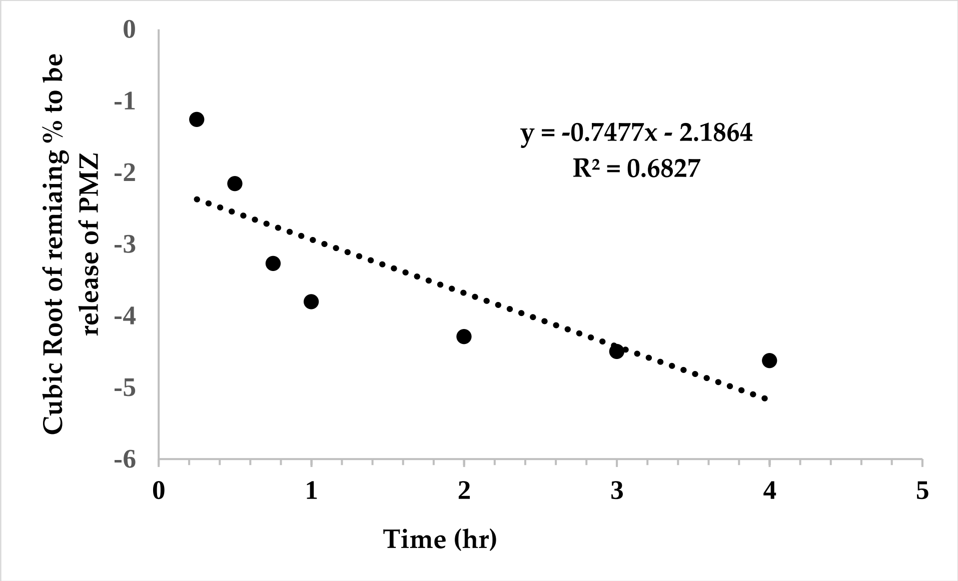

Figure S6. Linear regression of the Hixon Crowell kinetic mathematical model of PMZ from PMZ/Arg-PA NCs (F6). (R^2 is the coefficient of determination)

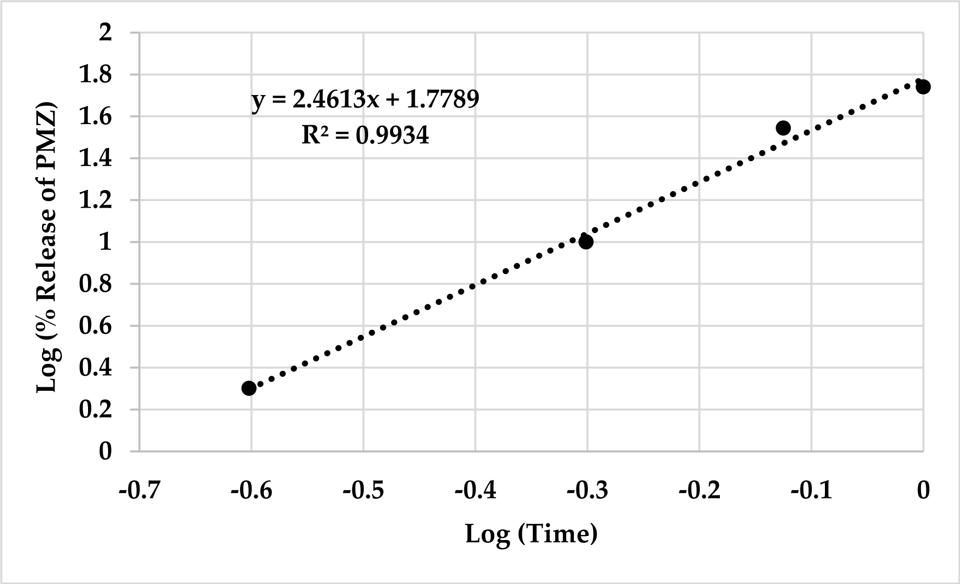

Figure S7. Linear regression of the Korsmeyer -Peppas kinetic mathematical model of PMZ from PMZ/Arg-PA NCs (F6). (R^2 is the coefficient of determination). Note this model requires the use of release data till 60% of material
